# From Infantilizing to Collaborating: Interactions in an Adult Day Center in Taiwan

**DOI:** 10.1093/geroni/igaa057.185

**Published:** 2020-12-16

**Authors:** Shumin Lin

**Affiliations:** National Chiao Tung University, Hsinchu, ROC, Taiwan (Republic of China)

## Abstract

Many studies have found that interactions in long-term care settings are characterized by infantilizing speech towards older adults, which was principally interpreted as detrimental to older adults’ health and self-esteem. These studies, however, focused on how caregivers talked to older adults and were conducted primarily in Western countries. How older adults respond to and make sense of such speech has received little empirical investigation (Marsden & Holmes, 2014). In this paper, I re-examined issues related to infantilizing speech based on 6 months of fieldwork in an ADC in Taiwan, which serves 33 older adults (aged 66-94), including 16 diagnosed with dementia. My data (including observational fieldnotes, 72 hours of video-recordings of naturally-occurring interactions, and conversations/interviews with caregivers, older adults and their family members) show that the ADC was discursively co-constructed as a learning place with frequent didactic interactions that occurred both ways. Many older adults (those with dementia included), with little or no education before, cherished the opportunity to be “students” for the first time. Caregivers also appreciated learning various things from the older adults. Furthermore, didactic interactions co-occurred or were interspersed with relational interactions, including teasing, humor, and bodily interactions that show mutual friendliness and care. By taking into account the wide variety of interactions, attending to the contributions of all parties, and situating these interactions in the personal as well as social histories, this study demonstrated that even didactic or so-called infantilizing interactions were used by caregivers and older adults as they collaborated to create strong positive relationships.

